# Preterm birth, birth weight, infant weight gain and their associations with childhood asthma and spirometry: a cross-sectional observational study in Nairobi, Kenya

**DOI:** 10.1136/bmjresp-2023-001895

**Published:** 2023-09-21

**Authors:** Helen Meme, Evans Amukoye, Cressida Bowyer, Jeremiah Chakaya, Ruaraidh Dobson, Jonathan Fuld, Cindy M Gray, Richard Kiplimo, Maia Lesosky, Kevin Mortimer, Amos Ndombi, Angela Obasi, Fred Orina, Jennifer K Quint, Sean Semple, Sarah E West, Lindsey Zurba, Graham Devereux

**Affiliations:** 1 Centre for Respiratory Disease Research, Kenya Medical Research Institute, Nairobi, Kenya; 2 Research and Development, Kenya Medical Research Institute, Nairobi, Kenya; 3 Faculty of Creative and Cultural Industries, University of Portsmouth, Portsmouth, UK; 4 Institute for Social Marketing and Health, University of Stirling, Stirling, UK; 5 Respiratory Medicine, Cambridge University Hospitals NHS Foundation Trust, Cambridge, UK; 6 School of Social and Political Sciences, University of Glasgow, Glasgow, UK; 7 Department of Clinical Sciences, Liverpool School of Tropical Medicine, Liverpool, UK; 8 Department of Medicine, University of Cambridge, Cambridge, UK; 9 Department of Medicine, College of Health Sciences University of KwaZulu-Natal, Cambridge, UK; 10 Department of International Public Health, Liverpool School of Tropical Medicine, Liverpool, UK; 11 Axess Sexual Health, Liverpool University Hospitals NHS Foundation Trust, Liverpool, UK; 12 NHLI, Imperial College London, London, UK; 13 Department of Environment and Geography, University of York, York, UK; 14 Education for Health Africa, Durban, South Africa

**Keywords:** asthma, asthma epidemiology, paediatric lung disaese

## Abstract

**Background:**

In sub-Saharan Africa, the origins of asthma and high prevalence of abnormal lung function remain unclear. In high-income countries (HICs), associations between birth measurements and childhood asthma and lung function highlight the importance of antenatal and early life factors in the aetiology of asthma and abnormal lung function in children. We present here the first study in sub-Saharan Africa to relate birth characteristics to both childhood respiratory symptoms and lung function.

**Methods:**

Children attending schools in two socioeconomically contrasting but geographically close areas of Nairobi, Kenya, were recruited to a cross-sectional study of childhood asthma and lung function. Questionnaires quantified respiratory symptoms and preterm birth; lung function was measured by spirometry; and parents were invited to bring the child’s immunisation booklet containing records of birth weight and serial weights in the first year.

**Results:**

2373 children participated, 52% girls, median age (IQR), 10 years (8–13). Spirometry data were available for 1622. Child immunisation booklets were available for 500 and birth weight and infant weight gain data were available for 323 and 494 children, respectively. In multivariable analyses, preterm birth was associated with the childhood symptoms ‘wheeze in the last 12 months’; OR 1.64, (95% CI 1.03 to 2.62), p=0.038; and ‘trouble breathing’ 3.18 (95% CI 2.27 to 4.45), p<0.001. Birth weight (kg) was associated with forced expiratory volume in 1 s z-score, regression coefficient (β) 0.30 (0.08, 0.52), p=0.008, FVC z-score 0.29 (95% CI 0.08 to 0.51); p=0.008 and restricted spirometry, OR 0.11 (95% CI 0.02 to 0.78), p=0.027.

**Conclusion:**

These associations are in keeping with those in HICs and highlight antenatal factors in the aetiology of asthma and lung function abnormalities in sub-Saharan Africa.

**WHAT IS ALREADY KNOWN ON THIS TOPIC**

- In high-income countries, the role of antenatal and early life factors in childhood asthma has been highlighted by numerous reports of associations between preterm birth, birth weight, infant weight gain and childhood asthma and lung function.

**WHAT THIS STUDY ADDS**

- For the first time in a sub-Saharan Africa setting, associations have been identified between birth characteristics and childhood symptoms consistent with asthma, and lung function measured by spirometry.

**HOW THIS STUDY MIGHT AFFECT RESEARCH, PRACTICE OR POLICY**

- These findings highlight the importance of antenatal factors in the development of asthma and lung function abnormalities in sub-Saharan Africa. Interventions that target the first 1000 days from conception are likely to be important for lifelong respiratory health in sub-Saharan Africa.

## Background

Asthma is the most common chronic condition of childhood. Although asthma prevalence rates in low-income and middle-income countries (LMICs) are lower than in high-income countries (HICs), it is acknowledged that the morbidity and mortality burden of asthma in LMICs is considerable.^1–4^ In recent years, high rates of abnormal lung function have been reported in sub-Saharan African countries with the reported prevalence of obstructed lung function ranging from 1.7% to 24.8%, (pooled prevalence 8%) and the reported prevalence of restricted lung function ranging from 18.5% to 72.0%.^5–8^

The first 1000 days, from conception to 2 years of age, is a critical window of growth and development^9^; and in HICs, it is generally accepted that exposures and lung development antenatally and during the first years of life are important in asthma aetiology and establishing lung function trajectories through life.^10 11^ Studies conducted in HICs investigating associations between childhood asthma/lung function and anthropometric parameters at birth/infancy have provided strong evidence for the importance of early life and stimulated much research in this area. Systematic reviews have reported that the likelihood of childhood wheezing and asthma is increased with preterm birth, low birth weight and higher infant weight gain (IWG).^12–17^ In meta-analyses of childhood lung function, forced expiratory volume in 1 s (FEV_1_) is negatively associated with preterm birth and low birth weight, but positively associated with IWG and the ratio FEV_1_/forced vital capacity (FVC) is lower in children born preterm, with low birth weight and higher IWG. Whereas meta-analyses and national cohort studies in HICs are numerous and include data from millions of participants,^14 15 17^ there have only been a handful of similar studies in LMICs^14^ especially sub-Saharan Africa where the rates of preterm birth and low birth weight are high.^18 19^ Small studies in Mozambique and Uganda of children hospitalised with asthma have inconsistently reported preterm birth (but not low birth weight) to be associated with asthma.^20 21^ In Ghana, birth weight has been reported to be inversely associated with airway resistance measured by oscillometry in 4-year-old children.^22^ There appear to be no studies in sub-Saharan Africa that have investigated, in a single study, preterm birth, birth weight, IWG, asthma and lung function measured by spirometry.

Tupumue (Swahili for ‘let us breathe’) is a cross-sectional observational study in Nairobi, Kenya, comparing the respiratory symptoms and lung function of schoolchildren within the informal settlement of Mukuru with schoolchildren in the nearby more affluent residential area of Buruburu.^23^ The study allowed us to investigate in a sub-Saharan African country whether birth weight, preterm birth, IWG are associated with childhood asthma and spirometry as reported in HICs.

## Materials and methods

The methods used in this cross-sectional study are described in detail elsewhere.^23^

### Setting

Kenya is a lower-middle-income country with a population of 55 million and per capita income of US$1816/year. The national rates of preterm (<37 weeks gestation) and low birth weight (<2500 g) births are 11.5% and 7.6%, respectively.^24 25^ The study was conducted in two areas of the capital city Nairobi. Mukuru is one of the largest informal settlements in Kenya, it is overcrowded and polluted, there is widespread poverty, poor sanitation and a lack of basic amenities. The nearby suburb of Buruburu is a large planned residential area that is mainly inhabited by business people and professionals.

### Patient and public involvement

The Tupumue study was codesigned in close co-operation with the two communities of Mukuru and Buruburu, with consultation meetings that included Chiefs, local politicians, parents, children, head teachers, teachers, social workers, counsellors, health workers and representatives from the Ministries of Education and Health.

To explain the study to the communities a suite of innovative, inclusive and culturally relevant sensitisation tools using the creative arts was cocreated by academics, arts practitioners, residents and community artists and delivered by local community ‘champions’ to audiences in schools, churches and community venues. All of the fieldworkers were recruited from the two communities.

### Recruitment

All children aged ≤18 years attending schools in Mukuru or Buruburu were eligible. Participating schools were randomly selected from a sampling frame of all schools. For each chosen school, a class from each year group was randomly selected.^23^

### Questionnaires

Field workers administered questionnaires to parents/guardians of children aged ≤12 years and to children aged ≥13 years.

The questionnaires included:

1. Demographics, school, age, sex, household asset-based wealth score.^26^
2. Respiratory symptoms.^27 28^
3. Parental/self-report of whether the study child was born preterm (<37 weeks of pregnancy).
4. Parental/self-report of environmental exposures, for example, exposure to traffic and domestic pollution sources including tobacco use.^28^

The questions asked are included in online supplemental material.

10.1136/bmjresp-2023-001895.supp1 Supplementary data

Parents were invited to bring the study child’s immunisation booklet. This booklet is distributed to all mothers in Kenya at the first contact of a newborn with a healthcare facility, it is used by healthcare providers to document encounters with health services, child immunisations and any illness. The page documenting birth weight and weight measurements made in the first year of life was photographed. Birth weights and serial weights were then entered into the study database.

### Spirometry

Children performed spirometry using the EasyOn Spirometer (NDD Medizintechnik AG, Switzerland) with on-screen incentive software. Nine technicians trained by Education for Health Africa and certified by the Pan African Thoracic Society (PATS) conducted spirometry in accordance with PATS, American Thoracic Society/European Respiratory Society (ATS/ERS) recommendations.^23 29^ Up to eight forced exhalation manoeuvres were performed while sitting and wearing nose clips. An internal and external assessor reviewed all blows, with measurements graded A–C for acceptability and repeatability being selected for analysis in accordance with PATS, ATS/ERS recommendations.^23 29^

### Air quality monitoring

Estimates of each child’s personal 24-hour exposure to fine particulate matter (PM_2.5_) were made by extrapolation from detailed air quality monitoring in the homes of 179 children, participating schools and outdoor settings.^23^ PM_2.5_ concentrations were measured using PurpleAir PA-II-SD sensors (PurpleAir, Draper, Utah, USA).

### Sample size

As described elsewhere, we aimed to collect symptom and spirometry data for 1000 and 800 children, respectively, from each of the two communities.^23^ For this study, we aimed to obtain data from as many child immunisation booklets as possible.

### Statistics

As in many epidemiological studies of asthma prevalence in children, asthma prevalence estimates were based on the responses to the questions ‘Has child had wheezing or whistling in the chest in the past 12 months?’(current wheeze), ‘Has child ever had asthma?’ (ever asthma), and ‘Has child used any inhaled medicines, for example, puffers to help his/her breathing problems at any time in the past 12 months?’ (asthma inhalers).^30 31^ Other respiratory symptoms of interest were cough (In the past 12 months, has this child had a dry cough at night, apart from a cough associated with a cold or chest infection?) and ‘trouble breathing’ (Does child ever have trouble with his/her breathing?). The spirometry parameters were FEV_1_, FVC and the ratio (FEV_1_/FVC) expressed as z-scores using Global Lung Initiative 2012 reference equations for African-American ethnicity in the absence of more appropriate Kenyan reference equations.^32^ The early life factors of interest were preterm birth, (identified by a positive response to the question: Was this child born prematurely (more than 3 weeks before he/she was expected)), birth weight and IWG (extracted from the child immunisation booklets, with IWG being calculated from weight measurements in the first year if there were ≥2 measurements in the first year ≥6 months apart).

Unadjusted and adjusted analyses were conducted. Adjusted analysis included potentially confounding covariates identified by literature review as being associated with wheezing outcomes and birth parameters (age, sex, household asset wealth score, community) which were available and measured and/or those environmental exposures significantly associated with outcomes in the primary analysis^23^ (exposure to vapours, dusts, gases or fumes >15 hours/week, smokers in the home, proximity of home to a major road, use of mosquito coils in the home). Hosmer-Lemeshow tests were used to confirm goodness-of-fit of logistic models. Analyses were performed using IBM SPSS Statistics for Windows, V.27.0.

## Results

Recruitment took place between January 2020 and November 2021 with a 14-month COVID-19 related suspension between March 2020 and April 2021. In total, 2373 schoolchildren were recruited; 1277 in Mukuru and 1096 in Buruburu. The characteristics of these children are presented in detail elsewhere and in online supplemental table S1.^23^ Briefly, when compared with Buruburu schoolchildren, Mukuru schoolchildren were (1 year) older, came from less affluent homes, were more likely to report being exposed to sources of air pollution in the home and had higher estimated PM_2.5_ exposures.^23^ More homes in Buruburu were close to major roads than in Mukuru (online supplemental table S1).^23^ The symptoms of ‘current wheeze’, and ‘trouble breathing’ were more likely to be reported in Mukuru, whereas ‘asthma’ was reported more frequently in Buruburu, there were no differences in the reporting of ‘dry nocturnal cough’ (online supplemental table S1). In total, 1655 children attempted spirometry, of which 1622 were acceptable and reproducible (98% of those attempting spirometry, 68% of total number recruited). A total of 718 children did not attempt spirometry, mainly because they could not be identified for spirometry testing post-COVID-19. As detailed elsewhere, although the children unable/unwilling to provide acceptable spirometry were younger (median difference 1 year), they were very similar demographically and symptomatically to those providing acceptable spirometry.^23^ There were no significant differences in spirometric parameters between Mukuru and Buruburu children.^23^

Tables 1 and 2 summarise the early life data collected during this study. Of the 2356 parents/children answering the question on preterm birth, 192 (8.1%) reported the study child was born preterm (Mukuru 8.7%, Buruburu 7.6%, p=0.326). Child immunisation booklets were provided by parents of 500 (21.1%) children, and birth weights were recorded for 323 children, the overall rate of low birth weight was 5.0%, (Buruburu 5.9%, Mukuru 2.9%, p=0.257). IWG data were extracted for 494 children. Children with child immunisation booklet data were more likely to be from Buruburu, be younger, from more affluent households and had less air pollution exposures (table 2). Although children with child immunisation booklet data had similar symptom and lung function profiles to those without child immunisation booklet data, they had a greater FEV_1_ z-score. Of the children with birth weight and IWG data from the child immunisation booklets, spirometry was available for 209 and 329 children, respectively.

**Table 1:** Availability and summary of early life data

	Buruburu	Mukuru	Combined
Preterm birth data (n)	1086	1270	2356
Preterm births (n, %)	82 (7.6)	110 (8.7)	192 (8.1)
Child immunisation booklet data	306	194	500
Birth weight data (n)	221	102	323
Birth weight (g), mean 95% CI	3205 (3137 to 3272)	3491 (3358 to 3624)	3295 (3232 to 3359)
Low birth weight (<2500 g) (n, %)	13 (5.9)	3 (2.9)	16 (5.0)
Infant weight gain data (n)	304	190	494
Infant weight gain (g/month, mean 95% CI)	517 (501 to 532)	547 (490 to 607)	529 (505 to 553)

**Table 2:** Socioeconomic, respiratory symptom and lung function characteristics of children with and without child immunisation booklet data

	Booklet data (n=500)	No booklet data (n=1873)	P value
Mukuru	194 (38.8%)	1083 (57.8%)	<0.001
Buruburu	306 (61.2%)	790 (42.0%)	
Girls (n, %)	252 (50.4%)	988 (52.7%)	0.350
Age (median, IQR)	9 (7–12)	11 (9–13)	<0.001
Household assets owned (median, IQR)	6 (3–7)	3 (2–3)	<0.001
Estimated 24-hour time weighted average PM2.5 (µg/m^3^) mean (95% CI)	30.0 (29.1 to 30.8)	32.2 (31.8 to 32.7)	0.004
Adults smoking in the home (n, %)	50 (10.0%)	199 (10.6%)	0.678
Symptoms			
Wheeze in last 12 months (n, %)	41 (8.2%)	149 (8.0%)	0.903
Trouble with breathing (n, %)	75 (15.0%)	271 (14.5%)	0.765
Dry cough at night in past 12 months (n, %)	72 (14.5%)	215 (11.6%)	0.074
Child ever had asthma (n, %)	11 (2.2%)	38 (2.0%)	0.811
Asthma inhalers in past 12 months (n, %)	2 (0.4%)	19 (1.0%)	0.192
Spirometry	Booklet data (n=333)	No booklet data (n=1289)	P value
FEV_1_ z-score mean (95% CI)	0.426 (0.326 to 0.527)	0.289 (0.230 to 0.348)	0.034
FVC z-score mean (95% CI)	0.354 (0.255 to 0.453)	0.265 (0.208 to 0.322)	0.156
FEV_1_/FVC z-score mean (95% CI)	0.103 (0.007 to 0.200)	0.035 (−0.017 to 0.088)	0.246

The questions asked are documented in online supplemental file.

FEV_1_, forced expiratory volume in 1 s; FVC, forced vital capacity; PM2.5, particulate matter.

In adjusted multivariable logistic regression models, preterm birth was positively associated with the symptoms of ‘current wheeze’, ‘trouble breathing’, and ‘dry nocturnal cough’ (table 3). There were no associations with birth weight or IWG, although the numbers included in the models were reduced. In adjusted linear regression models, birth weight was positively associated with FEV_1_ and FVC z-scores. IWG was negatively associated with FEV_1_/FVC z-score with a p value of 0.053. Further analyses including preterm birth in the models with birth weight made little difference in the magnitude of the estimates of the ORs/regression coefficients. Inclusion of FEV_1_, FVC, FEV_1_/FVC as absolute values with age, height and sex in the models made little difference to the findings apart from the association between IWG and FEV_1_/FVC percentage that achieved statistical significance, β=−3.80 (95% CI −7.12 to –0.48), p=0.025. Classifying spirometry into obstructed (FEV_1_/FVC<lower limit of normal, LLN) or restricted (FVC<LLN) indicated that 51 (3.1%) of children had obstructed and 39 (2.4%) had restricted spirometry and that birth weight was negatively associated with the odds of restricted spirometry OR 0.11 (95% CI 0.02 to 0.78), p=0.027.

**Table 3:** Multivariable linear and logistic regression modelling of respiratory symptoms and spirometry in relation to early life parameters

Symptoms	Preterm birth (n=2178)		Birth weight (/kg) (n=319)		Infant weight gain (/kg/month) (n=489)	
	OR* (95% CI)	P value	OR* (95% CI)	P value	OR* (95% CI)	P value
Wheeze in last 12 months	1.64 (1.03 to 2.62)	0.038	1.02 (0.53 to 1.97)	0.957	1.25 (0.45 to 3.51)	0.669
Trouble with breathing	3.18 (2.27 to 4.45)	<0.001	1.20 (0.70 to 2.05)	0.507	0.73 (0.25 to 2.16)	0.565
Dry cough at night in past 12 months	2.36 (1.63 to 3.41)	<0.001	1.63 (0.92 to 2.88)	0.092	1.83 (0.86 to 3.90)	0.120
Child ever had asthma	1.97 (0.85 to 4.55)	0.112	0.84 (0.20 to 3.58)	0.816	0.10 (0.00 to 16.5)	0.378
**Spirometry**	**Preterm birth (n=1603)**		**Birth weight (/kg) (n=209)**		**Infant weight gain (/kg/month) (n=329)**	
	β* (95% CI)	**P value**	β* (95% CI)	**P value**	β* (95% CI)	**P value**
FEV_1_ z-score	0.04 (−0.23 to 0.15)	0.681	0.30 (0.08 to 0.52)	0.008	0.10 (−0.56 to 0.75)	0.295
FVC z-score	0.02 (−0.20 to 0.17)	0.849	0.29 (0.08 to 0.51)	0.008	0.36 (−0.28 to 1.00)	0.267
FEV_1_/FVC z-score	0.09 (−0.26 to 0.09)	0.340	0.20 (−0.19 to 0.23)	0.182	0.61 (−1.23 to 0.01)	0.053

*Adjusted for: age, sex, asset wealth score, Mukuru/Buruburu, exposure to vapours, dusts, gases or fumes >15 hours/week, smokers in the home, proximity of home to a major road, use of mosquito coils in the home.

FEV_1_, forced expiratory volume in 1 s; FVC, forced vital capacity; β, regression coefficient.

## Discussion

In this observational cross-sectional study of children attending schools in two socioeconomically contrasting areas of Nairobi, Kenya, we report that preterm birth is associated with increased likelihood of the symptoms ‘current wheeze’, ‘trouble breathing’, ‘dry nocturnal cough’ and that birth weight is positively associated with FEV_1_ and FVC and reduced likelihood of restricted spirometry. We also highlight a negative association between IWG in the first year and FEV_1_/FVC ratio with a p value of 0.053. This is the first report of associations between preterm birth, birth weight and possibly IWG in relation to childhood symptoms consistent with asthma (current wheeze) and spirometry in a single sub-Saharan African setting and highlight the importance of early life factors in the aetiology of asthma and the life course of lung function in sub-Saharan Africa. These findings implicate the high rates of preterm birth, low birth weight and possibly poor IWG observed in sub-Saharan Africa as potentially important contributory factors to the prevalence of asthma, and obstructive and restrictive spirometry in sub-Saharan Africa.^1–8 18 24 25^

Studies in HICs with meta-analysis of very large numbers of children have reported: preterm birth, low birth weight and higher IWG are positively associated with wheezing and asthma; preterm birth, low birth weight and lower IWG are negatively associated with FEV_1_; and children born preterm, with low birth weight and higher IWG are more likely to have lower FEV1/FVC ratio.^12–17^ Although we were unable to demonstrate associations between birth weight, IWG and wheezing, or between preterm birth, IWG and FEV_1_ or between preterm birth, birth weight and FEV_1_/FVC, our findings with relation to wheezing, FEV_1_ and FEV_1_/FVC ratio from a relatively small study in a sub-Saharan country are entirely compatible with meta-analyses of studies conducted in HICs that are several orders of magnitude greater in size.

In sub-Saharan Africa, a few studies have related birth weight and preterm birth to early childhood respiratory outcomes, however, no single study in sub-Saharan Africa has investigated respiratory symptoms, spirometry, preterm birth, birth weight and IWG. In Mozambique, a case–control study of children aged 18 months to 8 years compared 100 children hospitalised with asthma to 99 age-matched controls, there was no association between low birth weight and asthma.^20^ In Uganda, a hospital-based cross-sectional study of 614 children aged 2–59 months admitted to hospital with respiratory complaints, preterm birth was associated with asthma (n=128) diagnosed by an expert panel (OR 9.3, 95% CI 1.2 to 83.3, p=0.044).^21^ The study we report differs from these because our study population was community rather than hospital based, older (4–18 years), we documented respiratory symptoms, lung function was measured and we used historical records of birth weight and serial infant weight measurements. The Ghana Randomised Air Pollution and Health Study study reported documented birth weight to be inversely associated with airway resistance measured by oscillometry in 567, 4-year-old children.^22^ Although this study was of children younger than those in the current study and did not collect symptom data, the association between birth weight and oscillometry is probably consistent with the association between birth weight and FEV_1_ reported in the current study. In South Africa, the Drakenstein child health study reported that birth weight z-score and gestational age at birth were associated with respiratory rate, functional residual capacity and respiratory system compliance measured in infants at 6–10 weeks of age.^33^ Although the infants studied were much younger than those in our study and there were no assessments of symptoms, the associations we report between birth weight and FEV_1_ and FVC are intuitively consistent with the associations reported in Drakenstein.

Further studies are needed to investigate whether antenatal interventions to increase birth weight and reduce premature birth, for example, maternal nutrition, air pollution exposure, reduce the risk of childhood asthma and improve childhood lung function. The limited data to date suggest that preterm birth and birth weight are not on the causal pathway to lung disease but reflect exposure to factors that influence not only fetal growth but also, to a variable extent, lung development/disease. In Nepal, a multiple micronutrient supplement during pregnancy increased birth weight but had no effect on childhood lung function or asthma,^34^ conversely, nutrient supplementation during pregnancy has been shown to increase childhood FEV_1_ and FVC in the absence of any effect on the rate of preterm birth.^35 36^

The strengths of the current study include the number of schoolchildren recruited, the use of widely accepted methodologies to investigate childhood asthma, that spirometry was conducted to international standards and that we were able to adjust for the effects of several potential confounding environmental exposures that we have shown to be associated with respiratory outcomes in this setting.^23^ Particular efforts (including the use of creative arts) were taken to ensure strong involvement of both communities. Unusually for a study in a sub-Saharan setting, we were able to use records of birth weight and serial measurements of weight made during infancy because we asked parents to bring their child’s child immunisation booklet. A limitation of our study is that child immunisation booklet data were only available for a minority of children (21%) and the records were incomplete, notably for birth weight. A number of biases were evident in parents providing child immunisation booklets, these probably relate to the younger age of the children and being from the more affluent Buruburu area. We observed that documented birth weights were higher in the informal settlement, however this is consistent with the findings of the 2012 Nairobi Cross-sectional Slums Survey.^37^ A further consequence of the limited availability of child immunisation booklet data was that some of the analyses conducted, most notably those relating birth weight to spirometric parameters, included relatively small numbers (n=209–489) of participants. A further limitation was our reliance on self-reported preterm birth (<37 weeks) instead of a recorded gestational age at delivery. Although we were not able to express birth weight adjusted for gestational age, we were able to adjust associations with birth weight for preterm birth. Throughout the study it was evident that both communities were aware of, and understood the terms asthma and wheeze, possibly reflecting the high English proficiency rate in Kenya and that English is an official language, however it remains a possibility that linguistic issues may have led to under-reporting of wheeze.^38^

The further limitation of the study is that we can only report associations because of the cross-sectional study design, and although we adjusted for potential confounding factors already identified we cannot exclude the possibility of residual confounding by unmeasured or mismeasured exposures, for example, parental asthma/allergic disease and maternal factors related to the study child’s pregnancy—nutrition, body mass index, age and parity. A further limitation is that we were particularly reliant on reports of respiratory symptoms instead of the metric ‘doctor diagnosed asthma’ that is a widely used and accepted measure of asthma prevalence in HICs. In sub-Saharan Africa ‘doctor diagnosed asthma’ is less reliable because asthma is underdiagnosed and undertreated,^3^ and our use of self/parental-reported ‘asthma’ is likely to be a significant underestimate of true asthma prevalence because parents are not aware of, or are reluctant to acknowledge that their children have asthma because of widespread underdiagnosis, misconceptions and stigma.^39^ A further limitation of the use of ‘ever asthma’ is that this may include children with preschool wheeze that may have resolved by the time the participants were studied at school age. Despite these limitations, the findings of the study were entirely consistent with those reported in HIC.

## Conclusions

In the first study in sub-Saharan Africa to relate birth characteristics to both childhood respiratory symptoms and lung function, associations have been demonstrated between: preterm birth and respiratory symptoms; between birth weight and FEV_1_, FVC and possibly IWG and FEV_1_/FVC. These findings are in keeping with those reported in HICs and highlight the importance of antenatal factors in the aetiology of asthma and lung function abnormalities in sub-Saharan Africa. Interventions that target the first 1000 days from conception have the potential for important benefits for lifelong respiratory health in sub-Saharan Africa.

## Data Availability

Data are available on reasonable request. Individual deidentified participant data that underlie the results reported in this article, along with data dictionary and study protocol are available on reasonable request. Please contact the corresponding author.
