# Supplementary material for: Preterm birth, birth weight, infant weight gain and their associations with childhood asthma and spirometry: a cross-sectional observational study in Nairobi, Kenya
Source: BMJ Open Respir Res. 2023 Sep 21;10(1):e001895. doi: 10.1136/bmjresp-2023-001895 (PMC10514609; doi:10.1136/bmjresp-2023-001895)
Supplement: Supplementary data [file bmjresp-2023-001895supp001.pdf]

Supplemental Material

Preterm birth, birth weight, infant weight gain and their associations with childhood asthma and spirometry: a cross-sectional observational study in Nairobi, Kenya.

Meme H, Amukoye E, Bowyer C, Chakaya J, Dobson R, Fuld J, et al

Meme H, *et al. BMJ Open Respir Res* 2023; 10:e001895. doi: 10.1136/bmjresp-2023-001895

Questions reported in the manuscript.

1. In the home where this child lives is there

2.	electricity,	(Y, N)
3.	a television,	(Y, N)
4.	a cell phone,	(Y, N)
5.	a refrigerator,	(Y, N)
6.	an indoor bath or shower,	(Y, N)
7.	an indoor tap,	(Y, N)
8.	a flush toilet,	(Y, N)
9.	a washing machine,	(Y, N)
10.	a landline telephone.	(Y, N)
11.	Does the family own a car	(Y, N)

12. Has this child had wheezing or whistling in the chest in the past 12 months? (Y / N /DK)
13. Has this child ever had asthma? (Y / N /DK)
14. Was this child’s asthma confirmed by a doctor? (Y / N /DK)
15. In the past 12 months, has this child had a dry cough at night, apart from a cough associated with a cold or chest infection? (Y / N /DK)
16. Does this child ever have trouble with his/her breathing? (never, only rarely, repeatedly, but it always gets completely better, continuously, so that breathing is never quite right)
17. Was this child born prematurely (more than 3 weeks before he/she was expected)? (Y / N /DK)
18. How close is your child’s home to a major road? (a road that has lorries and/or regular buses/minibuses) (<100m, 100-500m, >500 m)
19. Does anyone smoke cigarettes or tobacco inside the building where your child sleeps? (Y / N /DK)
20. In your child’s everyday life, does he/she breathe in vapours, dusts, gases or fumes for more than 15 hours per week? (Y / N /DK)
21. Is there ever a fire to burn refuse (waste, rubbish) within sight of your home? (Y / N /DK)
22. Do you burn mosquito coils at home? (Y / N /DK

Meme H, *et al. BMJ Open Respir Res* 2023; 10:e001895. doi: 10.1136/bmjresp-2023-001895

Table S1: Socio-economic, environmental, respiratory symptom, and lung function characteristics of participating children attending schools in Mukuru and Buruburu

	Buruburu (n=1096)	Mukuru (n=1277)	Combined (n=2373)
Girls (n, %)	567 (51.7%)	673 (52.7%)	1240 (52.3%)
Age (median, IQR)	10 (8-12)	11 (9-13)	10 (8-13)
Household assets owned (median, IQR)	6 (3-7)	3 (2-3)	3 (3-6)
Estimated 24-hour time weighted average $PM_{2.5}$ ($\mu g/m^3$) mean (95% CI)	22.3 (22.1, 22.5)	39.9 (39.5, 40.2)	31.7 (31.3, 32.2)
Exposed vapours, dusts, gases, fumes >15hrs/week (n, %)*	565 (54.6%)	815 (70.3%)	1380 (62.9%)
Refuse burnt within sight of home (n, %)	330 (30.2%)	485 (38.1%)	815 (34.5%)
Proximity of home to major road (n, %)			
<100m	569 (51.9%)	554 (43.4%)	1123 (47.3%)
100-500m	397 (36.2%)	398 (31.2%)	795 (33.5%)
>500m	130 (11.9%)	325 (25.5%)	455 (19.2%)
Smoker in the home (n, %)	90 (8.2%)	159 (12.5%)	249 (10.5%)
Burn mosquito coils in home (n, %)	197 (18.0%)	342 (26.8%)	539 (22.7%)
**Symptoms**			
Wheeze in last 12 months (n, %)	70 (6.4%)	120 (9.5%)	190 (8.1%)
Trouble with breathing (n, %)	138 (12.6%)	208 (16.3%)	346 (14.6%)
Dry cough at night in past 12 months (n, %)	135 (12.4%)	152 (12.0%)	287 (12.2%)
Child ever had asthma (n, %)	33 (3.0%)	16 (1.3%)	49 (2.1%)
Asthma inhalers	17 (1.6%)	4 (0.3%	21 (0.9%)
**Spirometry**	Buruburu (n=750)	Mukuru (n=872)	Combined (n=1622)
FEV_1 z-score mean (95% CI)	0.289 (0.214, 0.363)	0.341 (0.270, 0.412)	0.317 (0.266, 0.368)
FVC z-score mean (95% CI)	0.238 (0.167, 0.308)	0.322 (0.252, 0.391)	0.283 (0.233, 0.332)
FEV_1/FVC z-score mean (95% CI)	0.075 (0.004, 0.146)	0.027 (-0.034, 0.089)	0.049 (0.003, 0.096)

The questions asked are documented in supplemental file

CI confidence interval
